# Time-Frequency Analysis of Somatosensory Evoked High-Frequency (600 Hz) Oscillations as an Early Indicator of Arousal Recovery after Hypoxic-Ischemic Brain Injury

**DOI:** 10.3390/brainsci13010002

**Published:** 2022-12-20

**Authors:** Ze Ou, Yu Guo, Payam Gharibani, Ariel Slepyan, Denis Routkevitch, Anastasios Bezerianos, Romergryko G. Geocadin, Nitish V. Thakor

**Affiliations:** 1 Department of Biomedical Engineering, Johns Hopkins University School of Medicine, Baltimore, MD 21205, USA; 2 Departments of Neurology, Division of Neuroimmunology, Johns Hopkins University School of Medicine, Baltimore, MD 21205, USA; 3 Departments of Electrical and Computer Engineering, Johns Hopkins University, Baltimore, MD 21218, USA; 4 Information Technologies Institute (ITI), Center for Research and Technology Hellas (CERTH), 57001 Thessaloniki, Greece; 5 Departments of Neurology, Anesthesiology, Critical Care Medicine and Neurosurgery, Johns Hopkins University School of Medicine, Baltimore, MD 21287, USA

**Keywords:** somatosensory evoked potentials, high-frequency oscillations, cardiac arrest, continuous wavelet transform, hypoxic-ischemic injury

## Abstract

Cardiac arrest (CA) remains the leading cause of coma, and early arousal recovery indicators are needed to allocate critical care resources properly. High-frequency oscillations (HFOs) of somatosensory evoked potentials (SSEPs) have been shown to indicate responsive wakefulness days following CA. Nonetheless, their potential in the acute recovery phase, where the injury is reversible, has not been tested. We hypothesize that time-frequency (TF) analysis of HFOs can determine arousal recovery in the acute recovery phase. To test our hypothesis, eleven adult male Wistar rats were subjected to asphyxial CA (five with 3-min mild and six with 7-min moderate to severe CA) and SSEPs were recorded for 60 min post-resuscitation. Arousal level was quantified by the neurological deficit scale (NDS) at 4 h. Our results demonstrated that continuous wavelet transform (CWT) of SSEPs localizes HFOs in the TF domain under baseline conditions. The energy dispersed immediately after injury and gradually recovered. We proposed a novel TF-domain measure of HFO: the total power in the normal time-frequency space (NTFS) of HFO. We found that the NTFS power significantly separated the favorable and unfavorable outcome groups. We conclude that the NTFS power of HFOs provides earlier and objective determination of arousal recovery after CA.

## 1. Introduction

There are more than 356,000 out-of-hospital cardiac arrest (CA) occurrences annually in the U.S., with only 12.4% of patients surviving to hospital discharge [1]. CA remains the leading cause of coma, and there is a need for accurate neurological prognostication post-CA to properly allocate limited critical care resources [2,3,4,5]. However, most of the biomarkers developed, such as the bilateral absence of somatosensory evoked potential (SSEP) N20 peak, electroencephalogram (EEG) burst-suppression (BS) pattern, and high serum neuron-specific enolase, are all predictors of unfavorable neurological outcome [3,6,7,8,9]. Such interpretation could increase the risk of a “self-fulfilling prophecy”, i.e., if a variable is believed to suggest an unfavorable outcome, the belief will ultimately become a reality [2,10]. Therefore, we contend that the goal of research in this field is to find early predictors of favorable neurological outcomes that would help clinicians strategically allocate critical care resources while the hypoxic-ischemic injury is still reversible [11,12].

Somatosensory evoked potentials (SSEPs) are one of the commonly used modalities for post-CA prognostication [6,7,8], and there have been efforts to extract SSEP biomarkers to predict favorable neurological outcomes [13,14,15]. However, none of these efforts directly evaluated the integrity of the thalamocortical pathway, which is especially vulnerable to hypoxic-ischemic insults and is crucial for arousal [16,17,18,19,20]. In these studies [13,14,15], SSEPs are seen as a low-frequency phenomenon, but SSEPs also contain a series of low-amplitude high-frequency oscillations (HFOs) with a center frequency of 600 Hz superimposed onto the short-latency SSEPs [21]. Source dipole studies in humans and intracellular recording studies in mammalian animals have shown that HFOs contain two components separated by the N20 peak in humans (N10 in animals) [21,22,23,24,25,26]. The early HFO component is hypothesized to be generated by thalamocortical axon terminals in the primary somatosensory cortex (S1), and the late HFO component is hypothesized to be produced by highly synchronized spiking activities in S1. Several studies have investigated the connection between HFOs and arousal. Yamada et al. and Halboni et al. discovered that sleep attenuates HFO amplitude [27,28]. Gobbelé et al. found that eye opening/closing modulates HFO peak-to-peak amplitude in awake human subjects [29]. Restuccia and Coppola found that auditory stimulation elevates early HFO amplitude [30].

Despite the connection between HFOs and arousal, the application of HFOs to determine early and objective biomarkers of arousal recovery post-CA remains limited. Endisch et al., in a study of 302 comatose patients after CA, discovered that the late HFO component amplitude above 70 nV could faithfully eliminate the possibility of unresponsive wakefulness [26]. However, because this was a clinical study, the HFOs were recorded 24 h to 4 days after CA, missing the acute recovery period [31]. The acute recovery period, defined as the first four hours post-CA, is critical, because ischemic neuronal damage begins soon after the cessation of blood flow to the brain, and could still be reversible [14,32]. Previously, in an exploratory study on rats, we found that the HFO amplitude recovers earlier than the N10 amplitude in the acute recovery period and is associated with long-term neurological outcomes [33]. However, only the time-domain parameter peak-to-peak amplitude was considered for analysis [26,33]. Other previous studies have shown that the time-frequency (TF) domain representation of SSEPs can detect neurological injuries faster and more reliably than amplitude [34,35,36]. Nonetheless, TF domain parameters of HFOs have not been extensively tested for detecting not only neurological injury but also arousal recovery.

This study proposes the continuous wavelet transform (CWT) as a TF analysis method for HFOs in the acute recovery phase and explores the relationship between TF parameters of HFOs and arousal level quantified by the neurological deficit scale (NDS). We hypothesize that TF analysis of HFOs can indicate arousal recovery in the acute recovery phase. We first showed that CWT localizes HFOs as a single peak component in the TF domain. We then extracted HFO parameters from the TF domain and showed that the HFO TF measures could determine neurologic injury level. Lastly, we showed that HFO TF measures in the acute recovery phase exhibited significant separation between the favorable and unfavorable outcome groups defined by NDS evaluated at 4 h post-resuscitation.

## 2. Materials and Methods

### 2.1. Animal Model and Ethics

All experiment protocols were approved by the Johns Hopkins Animal Care and Use Committee. All animals were housed in a quiet environment with a 12 h day/night cycle and free access to food and water. The CA model used in this study has been validated previously [37,38,39].

Six male Wistar rats were subjected to 7 min asphyxial CA (group CA7) as the moderate to severe hypoxic-ischemic injury group, and five were subjected to 3 min asphyxial CA (group CA3) as the mild injury group. Our previous study showed that a minimum of five rats per group was sufficient to detect brain-injury differences [40].

### 2.2. Asphyxial CA Model and Experimental Procedures

**Figure 1:**
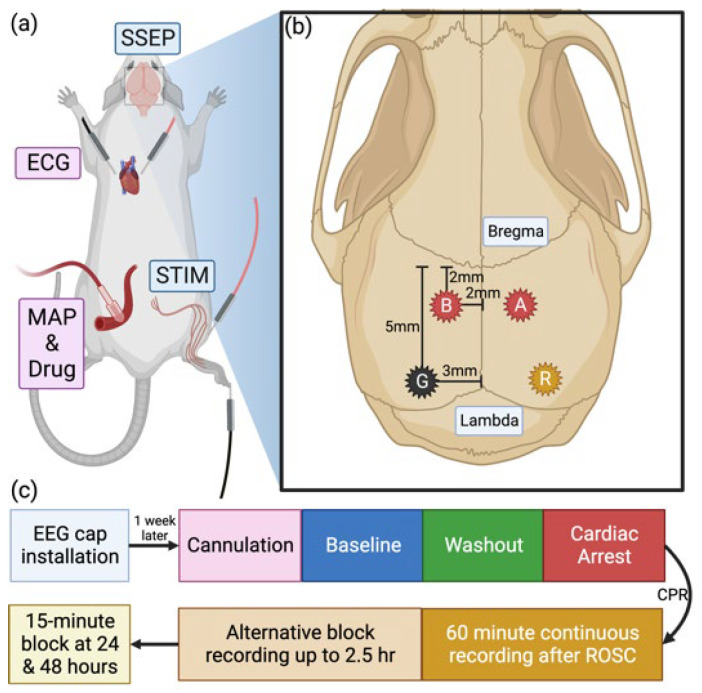
Experiment protocol. (**a**) Placements for arterial catheter, somatosensory evoked potential (SSEP) stimulating electrodes, and electrocardiogram (ECG) recording electrodes. (**b**) electroencephalogram (EEG)/SSEP recording electrode placements. Electrodes A and B were placed on the primary somatosensory cortex hindlimb regions (anterior/posterior (AP): −2; midline/lateral (ML): ±2). Ground (G) and reference (R) electrodes were placed on top of the posterior parietal lobe (AP: −5; ML: ±3). (**c**) Experimental timeline. Created with Biorender.com.

The asphyxial CA protocol has been previously described in detail (Figure 1) [14,39,41]. Briefly, the electrode implantation was performed 7–10 days prior to the asphyxia-induced CA experiment to ensure a full recovery. On the day of the CA experiment, the animals were endotracheally intubated and ventilated with 50%:50% N_2_/O_2_ containing 1.8% isoflurane (Piramal Critical Care, West Drayton, UK). The left femoral artery and vein were cannulated to record blood pressure and administrate medications, respectively.

After cannulation, a 10-min baseline recording of SSEPs was collected under 1.2% isoflurane. Following this, a 5-min anesthesia washout ensued, containing 2 min of 100% oxygen and 3 min of 80%:20% N_2_/O_2_. At the end of the 2 min of pure oxygen, rocuronium bromide (2 mg/kg, I.V.; Hospira, Inc., Lake Forest, IL, USA) was administered to induce muscle paralysis. After the washout period, asphyxia was initiated by stopping the ventilator and clamping the gas supply tube. After 3 or 7 min of asphyxia, ventilation was restarted with 100% oxygen. Cardiopulmonary resuscitation (CPR) was performed with sternal chest compression and injection of epinephrine (10 µg/kg, I.V.; PAR Sterile Products, Rochester, MI, USA) and sodium bicarbonate (1 mmol/kg, I.V.; Hospira, Inc., Lake Forest, IL, USA) until the return of spontaneous circulation (ROSC), defined as a mean arterial pressure (MAP) greater than 50 mmHg. Arterial blood gas was checked before and after CA. Following signs of regaining consciousness, the animals were sedated with 0.6% isoflurane. On average, anesthesia was reintroduced at 10 min post-ROSC for group CA3 and at 60 min post-ROSC for group CA7.

### 2.3. SSEP Stimulation and Recording Protocol

SSEPs were recorded using the Tucker-Davis Technologies (TDT) System3 data acquisition system (Tucker-Davis Technologies, Alachua, FL, USA). For the stimulation, a stainless-steel electrode (F-E2-48, Natus, Gort, Ireland) was inserted into the gap between the second and the third digits of the right hind paw, and another electrode was inserted into the center of the gastrocnemius muscle (Figure 1a). The existence of light muscle twitches confirmed the correct electrode placement. Stimulation pulses were 200 µs in duration and 1 mA in amplitude, and a single pulse was delivered every two seconds through an isolated constant current stimulator (DS3, Digitimer Ltd., Hertfordshire, UK) [41]. Instead of stimulating at 3 Hz, the typical frequency used in the clinics, we selected a lower frequency of 0.5 Hz to minimize animal discomfort, as limited anesthetic was used post-ROSC [14,41].

A week before the CA experiment, the animals were implanted with four transcranial screw electrodes (E363/20, P1 Technologies, Roanoke, VA, USA) for recording: two over the S1 hind limb region (anterior/posterior (AP): −2 mm; midline/lateral (ML): ±2 mm), and two over the posterior parietal lobe (AP: −5 mm; ML: ±3 mm) as ground and reference (Figure 1b). Electrodes made light contact with the dura mater without penetration into the brain. Electrodes, wires, and the exposed skull were covered by dental cement. The success of implantation and recovery was confirmed by baseline SSEP recording 1–5 days before the CA experiment.

SSEP signals were recorded with a sampling rate of 12.2 kHz. Each trial was recorded from 100 ms before to 200 ms after stimulation onset. SSEPs were recorded continuously for 10 min of baseline, 5 min of anesthesia washout, 3 or 7 min of CA, and one-hour post-ROSC. Subsequently, SSEPs were recorded in 15-min blocks alternating with 15 min rest up to 2 h post-ROSC. Afterward, SSEPs were recorded in 15-min blocks at 24 and 48 h post-ROSC. The single-trial recordings were averaged 100 times to improve the signal-to-noise ratio.

### 2.4. Neurological Evaluation

Rats were evaluated with the neurologic deficit scale (NDS) assessments at 4, 24, and 48 h post-ROSC by two independent and experienced observers. The NDS has been validated as a neurological outcome measure in rats after global hypoxic-ischemic injury [40,42]. The NDS score ranges from 0 to 80, where 80 represents neurologically normal subjects, and 0 is assigned to subjects that died before the evaluation. The complete NDS evaluation form is presented in Appendix A Table A1. The subjects were categorized into the favorable (NDS ≥ 65) and unfavorable (NDS < 65) outcome groups based on their NDS score evaluated at 4 h post-ROSC, which we had used previously as a measure of arousal [39,43]. The threshold of 65 was chosen based on the median NDS scores [39]. This categorization resulted in a favorable outcome group of six rats and an unfavorable outcome group of five. Five subjects from group CA3 and one subject from group CA7 formed the favorable outcome group, and five subjects from group CA7 formed the unfavorable outcome group.

### 2.5. Continuous Wavelet Transform

The continuous wavelet transform (CWT) converts a one-dimensional signal in the time domain into a two-dimensional matrix of coefficients in the time-frequency domain, where each CWT coefficient represents the similarity between the signal and a mother wavelet [44]. The mother wavelet is stretched or compressed to different scales and is shifted to different locations to compare with each portion of the signal. Thus, each CWT coefficient reflects signal power at a certain time and frequency. The CWT of signal $x\left(t\right)$ can be expressed mathematically as:

(1) $$C\left(a,b;x\left(t\right),\psi \left(t\right)\right)=\int_{-\infty }^{\infty }x\left(t\right)\frac{1}{a}\psi^{*}\left(\frac{t-b}{a}\right)dt,$$

where *a* is the scale parameter, *b* is the translation parameter, $\psi \left(t\right)$ is the mother wavelet function, and * is the complex conjugate operator.

The choice of the mother wavelet function will affect the CWT coefficients because each option has a unique shape. For this study, the Morlet wavelet, frequently used for neuro-electrical signal analysis, was selected [45,46]. Morlet wavelet belongs to the family of analytic wavelets, which have complex mother wavelets and optimal joint TF energy concentration and, thus, are more suitable for time-frequency representation [47,48].

### 2.6. SSEP and HFO Parameter Extraction

**Figure 2:**
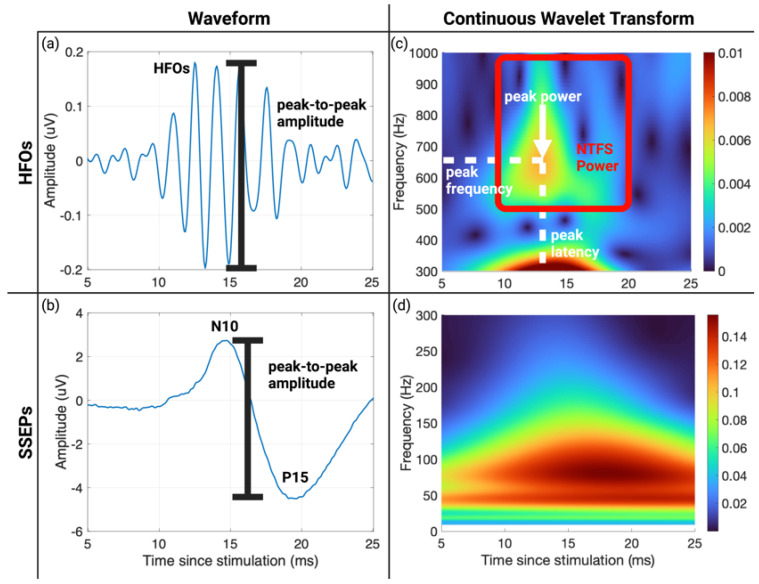
Parameter extraction from high-frequency oscillations (HFOs) and SSEPs in the time and time-frequency domain. (**a**) Grand averaged (*n* = 11 subjects) HFO waveform during anesthesia washout. The largest peak-to-peak amplitude was extracted as the parameter. (**b**) Grand averaged SSEP waveform during anesthesia washout. The N10-P15 peak-to-peak amplitude was extracted as the parameter. (**c**) Continuous wavelet transform (CWT) successfully isolated HFOs as a single peak around 600 Hz and 13 ms in the time-frequency domain. The color axis represents the signal power at a particular time and frequency. The peak latency, peak frequency, peak power, and the total power in the normal time-frequency space (NTFS) were used to characterize HFOs in the time-frequency (TF) domain. (**d**) SSEPs in the time-frequency domain using CWT. SSEP formed a single peak around 100 Hz and 20 ms. Created with Biorender.com.

The ipsilateral evoked potentials (electrode A) were subtracted from the contralateral evoked potentials (electrode B), equivalent to the clinical montage of C3′–C4′. The mean potential of the pre-stimulus baseline period in each trial was removed to form the single-trial SSEPs. To visualize SSEPs across subjects, the SSEP waveforms were normalized by the average amplitudes of the N10 and P15 peaks during washout to the range of [−0.5,0.5] for each subject. The N10-P15 peak-to-peak amplitude was extracted and normalized to its value during washout for each subject (Figure 2b).

The SSEPs were bandpass filtered between 500 Hz and 1000 Hz with a finite impulse response filter to isolate the HFO signal (Figure 2a). The cut-off frequencies were selected based on previous studies and visual inspection of the gap between HFOs and SSEPs (Figure 2c) [21,33,49]. The largest peak-to-peak amplitude of HFOs was extracted to compare with SSEP N10-P15 peak-to-peak amplitude (Figure 2a). Characteristics of the HFO peak of energy in the CWT TF domain were computed to quantify changes that may occur due to injury. The following parameters were computed: peak latency (measured relative to stimulation onset time), peak frequency, and peak power (calculated as the absolute value of the complex CWT peak coefficient). Furthermore, we defined the normal time-frequency space (NTFS) for HFOs as a time window from 10 to 20 ms post-stimulation and a frequency band from 500 Hz to 1000 Hz based on the time-frequency representation in Figure 2c. We then calculated the summation of the CWT coefficient absolute values in NTFS as the NTFS power (Figure 2c). Peak power and NTFS power of HFOs were normalized to their values during washout for each subject.

### 2.7. Statistical Analysis

The data were analyzed by MATLAB version R2021b (Mathworks, Natick, MA, USA). Wilcoxon rank sum tests were performed to compare the animals’ characteristics at baseline and the NDS scores of the two injury groups. Repeated measure analysis of variance (repeated measure ANOVA) was performed to test: if the SSEP N10-P15 peak-to-peak amplitude and the HFO peak-to-peak amplitude followed different trajectories of recovery; if the TF parameters of HFOs showed significant changes due to hypoxic-ischemic insult after CA; and if the time-domain peak-to-peak amplitude and TF-domain NTFS power could separate between the favorable outcome (NDS ≥ 65) and unfavorable outcome (NDS < 65) group during the first 60 min post-ROSC. The Shapiro–Wilk test was used to test the normality of the data, and Mauchly’s test of sphericity was used to test for the homogeneity of variance. If the assumption of sphericity was violated, the Greenhouse–Geisser correction was applied to the results of the repeated measure ANOVA. If the results of the repeated measure ANOVA tests were significant, post hoc analysis with Student’s t-test with Bonferroni correction was performed to compare distributions between every two sets of data. All reported *p*-values of post hoc analysis have been adjusted with the Bonferroni correction [50]. A *p*-value less than 0.05 was considered statistically significant. Data were presented as mean ± standard deviation unless noted otherwise.

To ensure the stability of the measures of HFOs, the within-subject coefficient of variance (*CV*) was calculated from the ratio of standard deviation to the mean value for HFO peak-to-peak amplitude, TF peak power, and NTFS power:

(2) $$CV=\frac{STD}{mean}.$$

Kruskal–Wallis tests were performed to compare the within-subject *CV*s of the three parameters during washout, CA, and recovery, followed by post hoc analysis with the Dunn test.

## 3. Results

Eleven rats were included in the analysis: Five were randomly selected for 3-min CA (group CA3), and the other six were randomly selected for 7-min CA (group CA7). Table 1 presents the animal characteristics and the NDS grouped by CA time. All rats in group CA3 survived to 48 h, and four out of six in group CA7 survived to that time. NDS scores were assessed at 4, 24, and 48 h post-ROSC. The NDS scores of group CA3 were better than group CA7 on all three occasions, and the difference was statistically significant at 4 and 24 h (*p* = 0.004 for 4 h; *p* = 0.016 for 24 h).

### 3.1. An Earlier Recovery of HFOs vs. SSEPs Shown in the Time Domain

**Figure 3:**
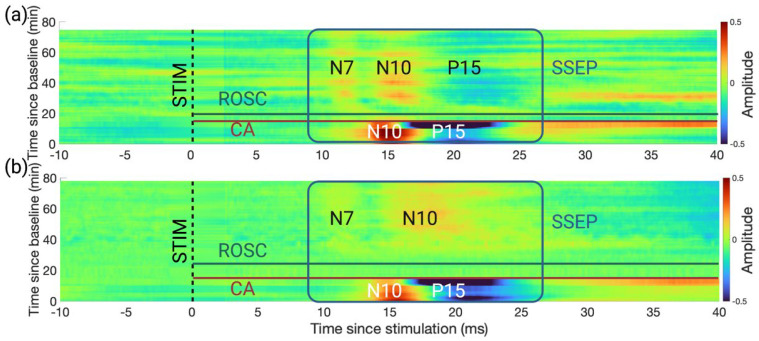
SSEP did not recover to the original waveform 60 min post-ROSC. (**a**) Evolution of SSEPs for group CA3 (*n* = 5 subjects). The *x*-axis spans from 10 ms before stimulation to 40 ms after stimulation. Each row represents one averaged SSEP waveform of 100 trials, and a twenty-second gap exists between subsequent rows. The *y*-axis spans from 0 min to 80 min since baseline. The onsets of CA and ROSC are highlighted with a red and a green line. The SSEP components are highlighted within the blue box. The N10 and P15 peak components were present before CA. During recovery, the N10 peak separated into two peaks, N7 and N10, and the P15 peak was preserved. (**b**) Evolution of SSEPs for group CA7 (*n* = 6 subjects) of CA. N10 separated into two peaks, N7 and N10, and the P15 peak was not preserved. Created with Biorender.com.

The broadband SSEPs followed the expected recovery trajectory after CA [41]. After a period of electrical silence immediately after CA, the short-latency SSEP components N7 and N10 appeared for both groups CA3 and CA7. The P15 peak was only preserved in group CA3. Figure 3 shows the grand average across-subject SSEP waveforms for the two injury groups as heatmaps.

**Figure 4:**
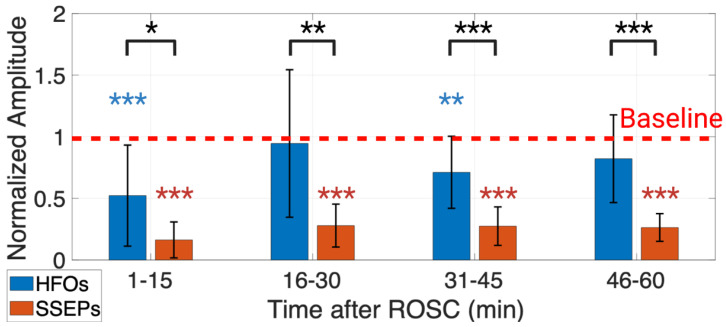
Normalized HFO peak-to-peak amplitudes recovered earlier and more robustly than normalized SSEP peak-to-peak amplitudes in the first 60 min post-ROSC (*n* = 11 subjects). The two-way repeated measure ANOVA test followed by a post hoc analysis found that the SSEP peak-to-peak amplitude was significantly different than baseline for the entire 60 min post-ROSC, and the HFO peak-to-peak amplitude was only significantly different for 1–15 min and 31–45 min post-ROSC. The difference between HFO and SSEP peak-to-peak amplitudes was statistically significant for all 60 min. * *p* < 0.05, ** *p* < 0.01, and *** *p* < 0.001. Created with Biorender.com.

HFOs experienced an earlier and more robust recovery than SSEPs according to their normalized peak-to-peak amplitude recovery (Figure 4). Peak-to-peak amplitudes of HFOs and SSEPs were normalized to their respective values during anesthesia washout. They were used to represent the percentage of recovery of HFOs and SSEPs, respectively. The two-way repeated measure ANOVA test showed that the interaction between signal type and experiment progression (*p* = 0.004) statistically affected normalized peak-to-peak amplitudes. The post hoc analysis found that while post-ROSC normalized HFO peak-to-peak amplitudes were only significantly different from baseline at 1–15 min and 31–45 min (*p* < 0.001 for 1–15 min; *p* = 0.009 for 31–45 min), post-ROSC normalized SSEP peak-to-peak amplitudes were significantly different from baseline for the entire 60 min (*p* < 0.001 for 1–60 min). In addition, the post hoc analysis found that the difference between the distribution of HFO and SSEP normalized amplitudes was statistically significant for the entire 60 min post-ROSC (*p* = 0.01 for 1–15 min; *p* = 0.002 for 16–30 min; *p* < 0.001 for 31–45 min; *p* < 0.001 for 46–60 min).

### 3.2. Evolution of HFO Recovery in the TF Domain

**Figure 5:**
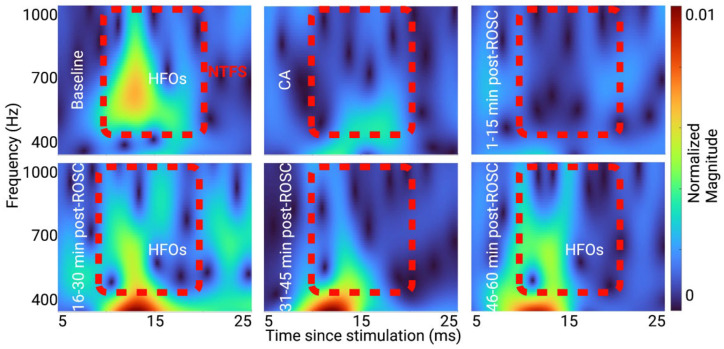
TF representation of grand average HFOs (*n* = 11 subjects) at washout, CA, and recovery periods 1–15 min, 16–30 min, 31–45 min, 46–60 min post-ROSC. CWT was used to transform the time-domain HFOs into their TF domain representations. The scalogram for each block, or the absolute value of the CWT coefficients of HFOs, was plotted. The color scale represents the absolute value of the CWT coefficients, i.e., the power of the HFOs at a particular time and frequency. The HFOs formed a single peak around 13 ms post-stimulation and 600 Hz in the TF domain during washout. However, the TF peak dissipated after CA and gradually recovered but did not form a single peak by the end of 60 min of recovery. In addition, a sudden burst of HFOs was present during the 16–30 min block. Created with Biorender.com.

The evolution of HFOs through injury is visualized clearly in the TF domain by CWT. Figure 5 shows the grand average (*n* = 11 subjects) absolute values of CWT coefficients for HFOs at washout, CA, 1–15 min, 16–30 min, 31–45 min, and 46–60 min post-ROSC. The absolute value of each CWT coefficient represents the energy of the HFO signal at a particular time and frequency. During washout, HFOs formed a single peak of energy in the time-frequency domain around 600 Hz and 15 ms. The concentrated energy dissipated after injury, experienced a sudden burst during 16 to 30 min post-ROSC, and gradually recovered. However, HFOs did not recover to a single peak in the TF domain after 60 min of recovery.

**Figure 6:**
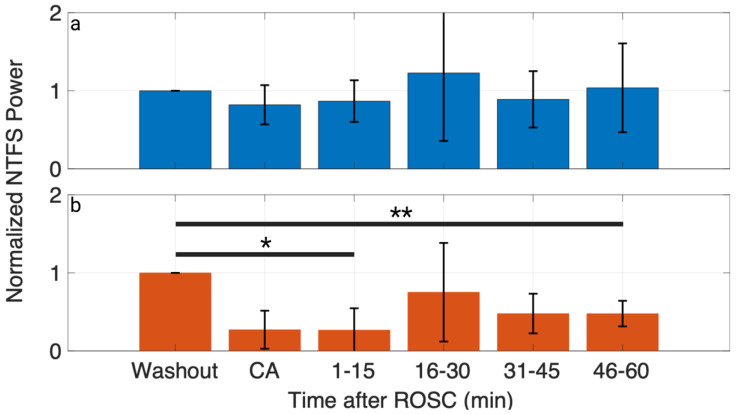
HFO NTFS power showed a significant decrease after 7 min of CA. (**a**) NTFS power evolution for group CA3 (*n* = 5). (**b**) NTFS power evolution for group CA7 (*n* = 6). * *p* < 0.05, ** *p* < 0.01. Created with Biorender.com.

To quantify the levels of injury to the HFO component, four time-frequency parameters of HFOs (peak latency, peak frequency, peak power, and NTFS power) were extracted from their CWT representation in the time window of 10 to 20 ms and the frequency window of 500 to 1000 Hz (Figure 2c). Peak latency, peak frequency, and peak power did not show significant changes for either group CA3 and CA7 (Appendix B Table A2), but repeated measure ANOVA test found that progression in the experiment had a statistically significant effect on HFO NTFS power for group CA7 (*p* = 0.027) (Figure 6). The post hoc analysis found that post-ROSC HFO NTFS power was significantly different from its washout level at 1–15 min (*p* = 0.02) and 46–60 min for group CA7 (*p* = 0.008).

### 3.3. NTFS Power of HFOs Associated with Neurological Outcome

**Figure 7:**
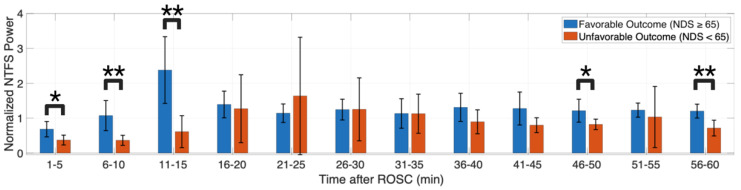
HFO NTFS power showed significant separation between favorable (*n* = 6) and unfavorable (*n* = 5) outcome groups during the first 60 min post-ROSC. * *p* < 0.05, ** *p* < 0.01. Created with Biorender.com.

**Figure 8:**
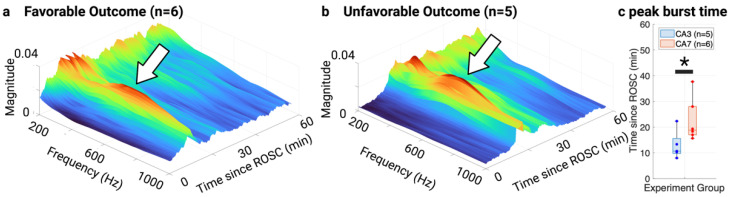
HFOs experienced a sudden burst post-ROSC for both outcome groups. The frequency spectrum of HFOs was plotted from 0 min to 60 min post-ROSC. The largest magnitude CWT coefficients for each frequency in the time window of 10 to 20 ms were extracted to represent the HFO spectrum. The left axis spans from 200 Hz to 1000 Hz; the right axis spans from 0 min to 60 min since ROSC; the vertical axis represents the magnitude of HFO at a particular frequency. (**a**) HFO spectrum for favorable outcome group. (**b**) HFO spectrum for unfavorable outcome group. (**c**) Peak burst time for both injury groups CA3 and CA7. * *p* < 0.05. Created with Biorender.com.

Lastly, we tested for the association between HFOs and neurologic outcomes quantified by NDS. We divided the subjects into a favorable and unfavorable outcome group using an NDS of 65 evaluated at 4 h post-ROSC as the partition. While the HFO peak-to-peak amplitude did not show a significant difference between the two outcome groups post-RSC (repeated measure ANOVA: *p* > 0.05), the HFO NTFS power did (*p* = 0.037). The post hoc analysis found that the difference between the two outcome groups was statistically significant at 1–5 min (*p* = 0.023), 6–10 min (*p* = 0.007), 11–15 min (*p* = 0.005), 46–50 min (*p* = 0.037), and 56–60 (*p* = 0.004) post-ROSC (Figure 7). However, the separation of NTFS power between the two outcome groups was not statistically significant from 16 to 45 min post-ROSC. The sudden HFO burst could partially explain the insignificance from 10 to 30 min post-ROSC (Figure 8a,b). However, the sudden burst time was associated more with the initial hypoxic-ischemic injury than neurological outcomes (Figure 8c). On average, the HFO burst peaked at 12.93 ± 5.58 min post-ROSC for group CA3 and 22.90 ± 7.69 min post-ROSC for group CA7, and a Wilcoxon rank sum test found the difference to be statistically significant (*p* = 0.03).

### 3.4. Stability of Time- and TF-Domain Measures of HFOs

To evaluate the stability of the time- and TF-domain measures of HFOs for post-CA neurological injury detection, the single-trial within-subject coefficients of variations (within-subject *CV*s) of time and TF parameters of HFOs were calculated for each block (Table 2). A Kruskal–Wallis test and post hoc analysis were performed to compare the within-subject *CV*s of the three parameters within each block. NTFS power was more stable with smaller within-subject *CV*s than HFO peak-to-peak amplitude during normal and injury conditions. The within-subject *CV*s of HFO peak-to-peak amplitude and TF peak power were similar. During washout, the within-subject *CV* of NTFS power was significantly lower than that of HFO peak-to-peak amplitude (Kruskal–Wallis *p* = 0.007; post hoc test *p* = 0.012).

## 4. Discussion

The current clinical need is to develop biomarkers that could objectively track recovery of arousal from coma that would help clinicians strategically allocate critical care resources [2,3,4,5]. While it has been shown that HFOs have the potential to be an indicator of responsive wakefulness days after CA in a clinical study [26], the evolution of HFOs in the acute recovery phase immediately after CA has not been adequately documented, which could be used to track arousal recovery and guide a treatment plan objectively. Therefore, this study utilized a well-established rodent CA model to characterize the evolution of HFOs in the acute recovery phase.

Using the traditional time-domain peak-to-peak amplitude measures, the experiments demonstrated that HFOs recovered earlier and more prominently than SSEPs in the acute recovery phase (Figure 4). Previous studies have found that the early portion of HFOs before the SSEP N10 peak has a thalamocortical origin [21,23,24]. Compared to the cortex, the thalamus is less vulnerable to hypoxic-ischemic injury after CA, as shown by neuroimaging and histology studies [51,52]. Thus, the partial thalamocortical origin of HFOs can be a factor in the early recovery of HFO peak-to-peak amplitude.

However, the time-domain peak-to-peak amplitude of HFOs did not show significant separation between the favorable and the unfavorable outcome groups during the acute recovery phase for the current study (repeated measure ANOVA: *p* > 0.05), and thus, indicates that it cannot objectively track the arousal recovery. Therefore, we explored TF-domain measures (peak latency, peak frequency, peak power, NTFS power) of HFOs.

The study showed that CWT could localize HFOs as a single peak in the TF domain during the baseline condition, but also showed that the peak of energy dissipated and partially recovered 60 min post-CA (Figure 5). Thus, we proposed NTFS power as a measure of how normal the HFO component is post-CA compared to baseline. For the current study, the NTFS for HFOs is defined as a time window from 10 ms to 20 ms post-stimulation and a frequency band from 500 Hz to 1000 Hz. Figure 2c shows a natural gap between the TF representations of HFOs and SSEPs from 400 to 500 Hz under baseline conditions. Similarly, Burnos et al. applied the Stockwell transform to SSEPs and showed a clear separation at 500 Hz between SSEPs and HFOs [49]. Thus, the passband of 500–1000 Hz was selected to quantify HFOs in the current study. Since the HFO energy is localized in the NTFS at baseline, a shift in time, frequency, or dispersion of the component would cause the NTFS power to decrease. Therefore, NTFS power summarizes any potential changes to HFOs into a single parameter. While peak latency, peak frequency, and peak power did not show significant differences compared to baseline (Appendix B Table A2), NTFS power did show a significant decrease post-ROSC compared to baseline for group CA7 (Figure 6).

To address the clinical need for early objective biomarkers of arousal recovery, the current study showed that the NTFS power of HFO showed a significant separation between the favorable and unfavorable outcome group while peak-to-peak amplitude did not (Figure 7). Endisch et al. evaluated the HFOs in comatose patients 24 h to 4 days after CA. They found that late HFO component peak-to-peak amplitude above 70 nV can safely exclude the possibility of unresponsive wakefulness or severe hypoxic-ischemic injury to the brain [26,31]. The result of the current study complemented the conclusion of Endisch et al. by showing that the separation of HFO activity between the favorable and unfavorable outcome groups began in the first 60 min post-ROSC. This result opens up the opportunity to further validate the use of NTFS power as an early biomarker of arousal recovery.

However, the separation between the two outcome groups was not significant for the entire duration of 60 min post-ROSC, partially due to the sudden HFO burst from 16 to 30 min post-ROSC (Table 2). The effect of this sudden increase in HFO energy is highlighted in Figure 8, where the frequency spectrum of HFOs was plotted for the 60 min post-ROSC. Previously, we found that EEG exhibited burst-suppression (BS) activity between isoelectric and continuous EEG after CA. BS activity appeared approximately 16 min after ROSC [53], coinciding with the period of HFO burst observed in the current study. Studies have suggested that the thalamus is at least partially involved in the generation of BS activity [54,55,56]. Thus, it is plausible that EEG BS activity and SSEP HFO burst post-ROSC share the same thalamocortical origin. Our future study will investigate the co-evolution of the two phenomena post-ROSC.

Because of the lower signal-to-noise ratio and non-stationarity of HFOs compared to SSEPs [49,57], it is essential to demonstrate that CWT does not introduce further noise to HFOs. Hu et al. have found that the TF-domain peak magnitude of SSEP has significantly lower within-subject variability than the time-domain peak amplitude [36,58]. A similar analysis was performed in the current study, and the result found that NTFS power has lower variability than peak-to-peak power under normal and injured conditions (Table 2). Although the improvement was only significant under the baseline condition, the result showed that it is not less reliable to quantify HFOs with NTFS power, while NTFS power provided the additional benefits of a two-dimensional representation of HFOs and significant separation between favorable and unfavorable outcome groups.

On the other hand, a few limitations to the current study require further investigation to develop NTFS power as an early biomarker of arousal. The sample size of the present study is relatively small. Although our previous study showed that a minimum of five rats per group was sufficient to detect the difference in brain injury [40], the use of NTFS power as an early biomarker of arousal needs to be further validated in larger cohorts. Furthermore, due to the limitation of our experimental setup, we only acquired unilateral SSEP from the right hindlimb. We believe that using bilateral will increase the power/accuracy of the proposed method in this paper.

## 5. Conclusions

TF analysis revealed that the energy peak of HFOs dispersed after CA, and we discovered the NTFS power of HFO as a reliable early biomarker for neurological injury and arousal recovery. The current work will lay the foundation for using TF analysis of HFOs as a novel neuromonitoring biomarker methodology for early arousal recovery from coma leading to functional recovery after CA.

## Figures and Tables

**Table 1:** Animal characteristics grouped by the duration of cardiac arrest (CA) (median [interquartile range]). NDS, neurologic deficit scale. ROSC, return of spontaneous circulation. CA3, 3-min CA. CA7, 7-min CA.

Characteristics	CA3 (*n* = 5)	CA7 (*n* = 6)	*p*-Value
Weight (g)	414 [30.75]	414 [74]	0.64
NDS at 4 h post-ROSC	80 [0.5]	58.5 [9]	0.004
NDS at 24 h post-ROSC	80.00 [0]	77 [27.75]	0.016
NDS at 48 h post-ROSC	80.00 [0]	78 [78.5]	0.21

**Table 2:** Within-subject coefficients of variations (*CV*s) of single-trial time and TF parameters of HFOs (mean ± SD).

Parameters	Washout	CA	Post-ROSC
Peak-to-peak-amplitude	48% ± 10%	42% ± 12%	59% ± 16%
TF peak power	47% ± 9%	41% ± 12%	60% ± 16%
NTFS power	35% ± 7%	33% ± 8%	55% ± 15%
*p*-Value	0.007	0.16	0.73

## Data Availability

The data presented in this study are available upon reasonable request from the corresponding author.

## Appendix A. Neurologic Deficit Scale

**Table A1:** Neurologic deficit scale for rats. (Normal condition = 80; worse outcome = 0).

Category	Score
General Behavior Deficit	0–19
Alerting	Normal (10), stuporous (5), comatose (0)
Eye opening	Open spontaneously (3), open to pain (1), absent (0)
Respiration	Normal (6), abnormal (3), absent (0)
Brainstem function	0–21
Olfaction	Present (3), absent (0)
Vision	Present (3), absent (0)
Pupillary reflex	Present (3), absent (0)
Corneal reflex	Present (3), absent (0)
Startle reflex	Present (3), absent (0)
Whisker stimulation	Present (3), absent (0)
Swallowing	Present (3), absent (0)
Motor Assessment	0–6
Strength	Normal (3), weak movement (1), no movement (0) (each side tested and scored separately)
Sensory Assessment	0–6
Pain response	Brisk withdrawal (3), weak movement (1), no movement (0) (each side tested and scored separately)
Motor behavior	0–6
Gait coordinate	Normal (3), abnormal (1), absent (0)
Balance beam walking	Normal (3), abnormal (1), absent (0)
Behavior	0–12
Righting reflex	Normal (3), abnormal (1), absent (0)
Negative geotaxis	Normal (3), abnormal (1), absent (0)
Visual placing	Normal (3), abnormal (1), absent (0)
Turning alley	Normal (3), abnormal (1), absent (0)
Seizures	0–10
Seizures	No seizure (10), focal seizure (5), generalized seizure (0)

## Appendix B. HFO TF Peak Latency, TF Peak Frequency, and TF Peak Power Evolution

**Table A2:** HFO TF peak latency, TF peak frequency, and TF peak power evolution (mean ± SD).

Parameters	CA Duration	Washout	CA	1–15 min	16–30 min	31–45 min	46–60 min	*p*-Value
Peak Latency (ms)	3 min	15.77 ± 4.26	13.70 ± 3.59	13.45 ± 3.68	12.26 ± 2.46	15.73 ± 3.49	12.59 ± 1.67	0.44
	7 min	16.05 ± 3.49	13.46 ± 2.68	15.70 ± 4.39	13.15 ± 3.67	12.67 ± 3.89	15.22 ± 3.82	0.18
Peak Frequency (Hz)	3 min	575 ± 90	627 ± 83	674 ± 143	612 ± 177	533 ± 50	540 ± 40	0.44
	7 min	563 ± 88	711 ± 235	729 ± 211	587 ± 107	573 ± 133	509 ± 14	0.18
Peak Power (normalized)	3 min	1.00 ± 0	0.69 ± 0.27	0.67 ± 0.23	1.19 ± 0.74	0.86 ± 0.32	0.98 ± 0.28	0.24
	7 min	1.00 ± 0	0.24 ± 0.23	0.26 ± 0.34	0.76 ± 0.80	0.56 ± 0.27	0.61 ± 0.23	0.05
